# A Letter from Paris

**Published:** 1875-04-01

**Authors:** C. C. Matteson

**Affiliations:** Paris


### Correspondence.

### A LETTER FROM PARIS.

EDITORS MEDICAL EXAMINER: Two women (Mrs. Garnet Anderson, of London, and Mrs. Putnam Jacobi, of New York,) have already received the degree of Doctor in Medicine from the French School, and this winter there are about a dozen women inscribed at the Faculty of Paris. Yet the profession in France does not agree as to the propriety of having women doctors so unanimously as might be supposed. Lately the *Union Medicale*, a journal occupying an influential position, has contained two articles upon "*femmes-medicins*," which are very marked in their opposition to woman's entrance upon a medical career. The writer, after enlarging upon the distaste with which one views a woman in the dissecting or post-mortem room, turns his attention to the practical life that follows upon medical studies: "And, in the exercise of the art, suppose the doctress reaches the eighth month of a pregnancy (a circumstance which will naturally be frequent if the doctresses are numerous), see this woman entering the bed-room of her patient, preceded by an enormous belly, approaching with difficulty to feel the pulse, to auscult in front and behind, an object of repugnance for the patient, if man or woman; an object of fright from her deformity, if the patient is a child. The spectacle would be grotesque, if it were not pitiful." Further on we find the following: "A young woman belonging to one of the most respectable families of Paris, mother of two children, reached her third pregnancy. From an unfounded sentiment of prudery she did not wish to receive the care of a physician during her confinement. She was assisted by a midwife. At the end of the labor, a grave uterine hæmorrhage declared itself. It became formidable. What did the woman-doctor do? She fainted! It was the way to relieve herself from all responsibility, but during that syncope the poor young mother perished. I ask, do we see accoucheurs, in the presence of an uterine hæmorrhage, faint, in place of arresting the flow? Syncope is in the nature of woman; it will be necessarily one of the attributes of a doctress."

In concluding the first article, the writer argues that woman was not intended for medical investigations: "No, she was not created to meditate upon a skeleton, to explain the state of our organs by percussion and auscultation, to plunge her small hands into the abdomen of the dead, and there search the cause of death." Mentioning the University of Zurich, the writer calls attention to the disturbances made by the female students (many of them were Russians), on account of which the Emperor of Russia issued a special decree, forbidding the right of practice in Russia for any woman with a Zurich diploma. It is related that one of the professors at that institution, having a "sore throat," requested the students not to smoke in the lecture-room. The next day the male students threw away their cigars before entering, but the females entered with immense pipes in their mouths, and smoked "*comme des caporaux*."

These reflections upon the character of women as medical students do not accord with my observations in Paris. I have yet to see one of them act in a way to attract attention. Though in no way approving their choice of a profession, I have been struck by the earnest, unassuming manner in which they conduct themselves. As a rule, they are attired very plainly in black, wearing little or no jewelry, but there is one rather pretty little woman, of perhaps twenty years (this is dangerous ground, so I must say, as the French do, "under all reserve,") who attends the lectures and demonstrations on practical anatomy, wearing the bright colors that best become her, but which form a striking contrast with the surroundings. Her entrance in the lecture room is generally acknowledged by some few suggestive "smackings" of lips, but thus far I have noticed no other manifestations from the students.

Something of a rivalry appears to exist at present among the eye specialists of Paris, in regard to dispensary service, and medical visitors are received with a great deal of attention. Desmarres (fils), Galezowski, and Wecker have the principal clinics. The latter has quite an elegant mansion for his free service on the Rue du Cherche-Midi, while the other two content themselves with less pretentious rooms on the Rue Hautefeuille and Rue Dauphine.

I notice that anæsthetics are used rather sparingly, and very many operations, even to enucleation of the ball, are performed without ether or chloroform. Undoubtedly, this is a neat mode and agreeable for the operator, but hardly in accordance with our American ideas of justice to the patient.

Solutions of atropia and the nitrate of silver occupy their usual prominent position in these eye clinics, but Galezowski has taken to operative interference in two diseases that are classically treated with caustics. I refer to chronic granulations of the lids and purulent ophthalmia. In obstinate or severe cases, he excises a small portion of the conjunctiva, and follows the operation with very weak solutions of nitrate of silver when the inflammation has subsided.

For the granulations, the excision is made in the cul-de-sac, from the palpebral conjunctiva, within or among the posterior granulations. Complete rest for a day or two, and then applications as mentioned (Argent. Nit. grs. i. ij.; Aquæ ℥ j.). With this line of treatment he claims to radically cure the most obstinate cases.

As to the ophthalmia, the operation is but changed in position. The excision is made in the orbital conjunctiva, and Galezowski's reasoning is, that the pus being harmless, the excision relieves the tension, and thus counteracts the tendency to perforation of the cornea. His results, thus far, have been very satisfactory.

Since Huxley exposed the fallacy of Bastian's experiments in regard to spontaneous generation, little has been attempted in that line; but now, so distinguished a body as the French Academy of Medicine finds itself called upon to investigate that subject. Dr. Arthur Bergeron has been examining, microscopically, the contents of abscesses that have never been in contact with the exterior air, and he found that the pus from adult subjects contained animalcules (protozoaires) while that from young subjects gave no trace of them. From these facts Dr. Bergeron concludes that he has made a great discovery, and that he has found the solution of the great question of spontaneous generation, "from simple modification of organic matter without intervention of germs or ferments."

M. Pasteur has brought these investigations before the Academy of Medicine, but at present, that body, not having been directly addressed by the investigator, is unable to appoint a committee of inquiry. Perhaps it is just as well that this unsurmountable barrier of dignity should prevent the Academy from giving much serious attention to this subject, but these researches are being pretty widely commented upon by the journals, and doubtless the question will be thoroughly ventilated.

C. C. MATTESON, M.D.

PARIS, Feb. 25th, 1875.

---

Dr. CAZIN has had occasion to practice the Cæsarian operation in a case of uterine fibroids, and has had the good fortune to save mother and child. He operated on a woman, aged thirty-nine, in whom, toward the sixth month of pregnancy, fibroid tumors were recognized in the posterior and inferior wall of the uterus. Labor set in the seventh month; after four days of pains, the waters ruptured and the hand escaped, the child still living; but as it could not be extracted either by forceps or by version, recourse was had to the Cæsarian operation. The most minute precautions were taken; there were hæmorrhage and syncope, inertia of the uterus, distention of the belly to such a degree that it became necessary to puncture the bowel to give exit to gas; there was vesical paralysis, and an abscess formed between the uterus and the abdominal wall. In spite of all these complications, the patient got well; and the child, baptized Cæsar, throve well. This operation was done some months ago, but the author has ascertained that the fibroids are in process of diminution.—*Gaz. Med.*

